# Management of Catheter-Related Urethral Injuries in Male Children

**DOI:** 10.7759/cureus.76405

**Published:** 2024-12-26

**Authors:** Gregory Harrison, Alice Pennington, Karim Awad

**Affiliations:** 1 Paediatric Surgery, Bristol Royal Hospital for Children, Bristol, GBR; 2 Urology, Royal London Hospital, London, GBR; 3 Paediatric Surgery, Ain Shams General Hospital, Cairo, EGY

**Keywords:** anterior urethra, catheter-related urethral injury, management algoirthm, patient follow-up, radiological investigations, urethral injury, urethral trauma, urethra perforation, urinary catheter

## Abstract

Introduction: Management of urethral trauma lacks clarity in the paediatric population. There is no clear guidance for management and follow-up of these patients which can lead to missing the long-term sequelae of the primary injury.

Catheter-associated urethral injuries are less likely to cause a complete transaction of the urethra. This is due to the mechanism, typically caused by creating a false passage or inflating the balloon in the urethra. In partial urethral injuries, the European Association of Urology (EAU) guidelines suggest follow-up after one-two weeks of bladder drainage or a urethrogram.

The purpose of this study was to review literature related to the management and follow-up of catheter-induced urethral injuries, subsequently comparing this to a case series in a single paediatric tertiary centre. The aim was to propose a unique algorithm to safely and effectively guide clinicians for this presentation.

Results: In our case series, 11 of 12 required initial bladder drainage. The data demonstrated an inconsistent approach to investigations throughout their admissions. Most cases had a successful trial without catheter (TWOC) or ability to resume continuous intermittent catheterisation. One patient needed a vesicostomy. We had a single bulbar urethral stricture, which wouldn't permit an 8fr catheter. This was managed using cystoscopy and serial urethral dilations.

Our cohort is likely an underrepresentation of the actual number of catheter-related injuries in our institute. Some injuries are managed by the parent team without referring to paediatric urologists if spontaneous micturition occurs or if they manage to catheterise after an initial traumatic attempt.

Conclusion: Catheter-related urethral injuries are common but underreported. They are less likely to have long-term sequelae than other mechanisms of trauma. The majority of cases do well following a period of initial bladder drainage. Current practise varies even in one institute as there are no clear management and follow-up guidance in current literature. Our proposed algorithm is a useful tool and decreases the incidence of missing long-term sequelae.

Management algorithm: Post urethral injury, a child who is passing urine with conservative management is likely to have good long-term function. They would require re-assessment after discharge. In clinic they would require urinary flow assessment and post-void residuals. If not toilet trained, parental impression of whether their child’s stream is interrupted or if they strain during urination would be assessed. Back-pressure changes would be considered on ultrasound scan (USS). If the assessment indicates concern, then a micturating cystourethrogram (MCUG) assessment for children younger than one or a cystoscopic assessment for children older than one would be recommended.

Post urethral injury, if a child is unable to pass urine conservatively, then an urgent urological assessment would be appropriate. An attempt at catheterisation would be made. If unsuccessful, the patient would be assessed for theatre. If unfit for it, an ultrasound-guided suprapubic (SP) catheter would be advised. If the patient is fit, then a cystoscopic and wire-guided catheter would be preferred. Later, if they passed a TWOC, they would be managed as per the algorithm described above. If they failed the TWOC, MCUG would be proceeded to. Catheter management and regular follow-up, or for a definitive intervention would be planned for.

## Introduction

Iatrogenic injury is the commonest cause of urethral trauma [1]. The incidence of male urethral injury during transurethral catheterisation is 13.4 per 1,000. Likely causes of injury include poor technique, creation of a false passage, inappropriate choice of catheter, lack of lubrication, catheter balloon inflation before or without urinary drainage, insufficient fixation of the catheter and traumatic removal of the catheter by the child [2].

Catheter-related traumas are most likely to cause lesions to the anterior urethra. The anterior urethra is defined as the fossa navicularis, penile and bulbar urethra. Management of urethral trauma lacks clarity in the paediatric population as it is an uncommon presentation. Research varies, promoting active and passive management, alongside delayed and immediate management. There is also debate about reliable diagnostic modality and using intraoperative, clinical or radiological evaluation. Principles when managing urethral injuries are to ensure safe urinary drainage and avoidance of long-term complications [3-12].

Acute risks of trauma to the urethra include urine extravasation at the site of injury and urinary retention. Chronic issues develop as periurethral diverticulae, urethrocutaneous fistulae, impotence, stricture formation and incontinence. These can cause significant life-long morbidity [13]. There is no clear guidance for management and follow-up of these patients which subsequently can lead to missing long-term sequelae of the primary injury.

Aim

The purpose of this study was to review literature related to the management and follow-up of catheter-induced urethral injuries, subsequently comparing this to a case series in a single paediatric tertiary centre. The aim was to propose an unique algorithm to safely and effectively guide clinicians for this presentation.

## Materials and methods

A literature review was performed using Google scholar, Pubmed, Embase and the Cochrane library. We searched for articles using a combination of the following: *"paediatric", "catheter" “urethral injury”, “false passage”, “urinary retention”, "stricture", "management", "treatment"* and synonyms of such words. This literature review was used to explain the benefit of a standardised management algorithm.

A case series in a single paediatric surgical centre was retrospectively analysed to assess the variation of care for catheter-related urethral injuries . Data was harvested from a locally maintained surgical database. All cases of paediatric urethral injury from 2007 to 2023 were included. This was achieved by searching for “*urethral injury*”, “*false passage*” and “*urinary retention*” in their case notes. Inclusion criteria of the case series were an iatrogenic urethral injury caused by the insertion of a catheter. Exclusion criteria were cases which had alternative traumatic causes of urethral injury or were not related to injury at all.

Cases identified were cross-checked using hospital software to confirm age at injury, cause of injury, initial investigations and management, definitive management, complications and long-term outcomes. This software included electronic medical records and picture archiving and communication system (PACS), providing access to inpatient notes, including operation notes, investigation requests and reports, and clinic letters. On two occasions, archived notes were retrieved for review. Microsoft Excel 2010 was used to organise and present the case series demonstrated in the results.

The study had a small cohort, hence interpretations from the data were not statistically assessed and this study was reported as a case series. 

The proposed management algorithm was created using our literature review, case series and modifying British Association of Urological Surgeons (BAUS) algorithms for management of traumatic urethral injuries [3].

## Results

Case series

We identified 12 patients with catheter-induced urethral trauma. All cases had iatrogenic urethral injury following traumatic urethral catheterisation. The patients were males aged 15 days to 14 years.

The study had a small cohort, hence interpretations from the data were not statistically assessed.

The findings are presented in Tables 1-4.

**Table 1:** Presentation of injury CIC: Clean intermittent catheterisation; MCUG: Micturating cystourethrogram

Presentation	Case frequency
Haematuria	4
Urinary retention and urethral bleeding	3
Urinary retention	1
Failure to resume previously established CIC due to false passage	3
Failure to catheterise for MCUG (Investigating antenatal hydronephrosis)	1

**Table 2:** Initial investigation All bladder USSs showed urinary retention and intravesical haematomas of variable sizes. USS: Ultrasound scan

Initial investigation	Case frequency
Nil	5
Cystoscopy	3
Retrograde urethrogram	1
USS	3

**Table 3:** Initial intervention SP: Suprapubic; USS: Ultrasound scan; PUV: Posterior urethral valves

Initial intervention	Case frequency
Insertion of urethral catheter by urologist	5
Insertion of SP catheter (via USS)	2
Cystoscopy + guidewire catheter insertion	2
Conservative	1
Fluroscopy guided insertion of catheter	1
Cystoscopy + stricture dilatation + PUV resection + catheter insertion	1

**Table 4:** Outcome of injury TWOC: Trial without catheter; MCUG: Micturating cystourethrogram; CIC: Clean intermittent catheterisation

Outcome	Case frequency
Successful TWOC	5
Successful TWOC + MCUG normal	2
Restarted CIC	2
Successful TWOC and serial dilatations of bulbar stricture	1
Vesicostomy as CIC couldn't be resumed after injury	1
Deceased from other cause	1

Follow-up post discharge

Patients did not have any follow-up post discharge in six cases and had follow-up in five. In one case, the patient was observed and voided spontaneously without the need for catheterisation. He died of other causes before long-term urological outcomes could be assessed.

The chronic patients who were on clean intermittent catheterisation (CIC) (two neurogenic bladder patients, one posterior urethral valves (PUV) and one hypoplastic urethra) were followed up with routine annual renal ultrasound scan (USS). The PUV patient needed check scopes and serial dilatations of his bulbar urethral stricture, which settled after four dilatations. One had symptoms of frequency and urgency, with poor bladder emptying on repeat renal USS and is currently on the waiting list for check cystoscopy.

## Discussion

Paediatric urethral injuries are considered rare traumas and it is not uncommon to lack sufficient expertise in a single centre [14]. The data published on paediatric catheter-related urethral injuries are even more scanty. Paediatric urethral trauma management has traditionally followed adult treatment algorithms due to the limited available paediatric specific data [15-17].

In the surgical treatment of urethral injuries, three different approaches are considered: early (within two days), delayed (two to 14 days), and late (after three months) [18]. In catheter-related injuries, they invariably lie into the late repair group if further intervention is required.

The general treatment approach for mild to moderate penile urethral injuries suggests conservative management, such as a suprapubic (SP) catheter or penile catheterisation for one to two weeks. This is due to the mechanism of trauma, typically caused by creating a false passage or inflating the balloon in the urethra. These scenarios are less likely to cause a complete transaction of the urethra and respond to a period of drainage [3,19,20].

Our cohort is demonstrated in Table 5. It shows that 11 patients (92%) required initial bladder drainage which was achieved by an SP catheter in two patients whilst the rest had a urethral catheter inserted either by a gentle trial by a urologist, fluoroscopy guided or cystoscopy guided. They all then had successful trials without catheter (TWOCs) or ability to resume CIC other than one patient who needed a vesicostomy for inability to resume CIC following a false passage creation by a catheter. Another case is being investigated currently due to reduced urinary flow. 

**Table 5:** Summary of the results TWOC: Trial without catheter; MCUG: Micturating cystourethrogram; USS: Ultrasound scan; SP: Suprapubic; CIC: Clean intermittent catheterisation

Patient	Presentation	Initial investigation	Initial management	Further management	Outcome	Follow up
1	Haematuria	None	Catheter insertion by urologist	Conservative	Successful TWOC	None
2	Haematuria	None	Catheter insertion by urologist	MCUG: Normal	Successful TWOC	Renal USS + uroflows + awaiting check cystoscopy
3	Haematuria	Bladder USS	Catheter insertion by urologist	MCUG: Normal	Successful TWOC	None
4	Haematuria	None	Conservative	None	Spontaneous voiding	Deceased
5	Urinary retention + urethral bleeding	None	Catheter insertion by urologist	Failed MCUG: Catheter tip coiled from bladder to posterior urethra	Successful TWOC	None
6	Urinary retention + urethral bleeding	Bladder USS	USS guided insertion of SP catheter	Conservative	Successful TWOC	None
7	Urinary retention + urethral bleeding	Bladder USS	USS guided insertion of SP catheter	Conservative	Successful TWOC	None
8	Urinary retention	Retrograde urethrogram	Fluroscopy guided catheter insertion	Conservative	Successful TWOC	None
9	Failed catheter insertion for MCUG	Cystoscopy	Cystoscopic dilatation of catheter-induced bulbar urethral stricture + resection of PUV + catheter insertion	Repeated dilatation for stricture (x4)	Successful TWOC	Annual renal USS
10	Failure to resume CIC	None	Catheter insertion by urologist	Vesicostomy formation	Failed to resume CIC	Annual renal USS
11	Failure to resume CIC	Cystoscopy	Cystoscopy + guidewire catheter insertion	Conservative	Restarted CIC successfully	Annual renal USS
12	Failure to resume CIC	Cystoscopy	Cystoscopy + guidewire catheter insertion	Conservative	Restarted CIC successfully	Annual renal USS

D’Cruz et al. have published their experience with managing six boys aged one month to 16 years old with catheter-related urethral injuries over a period of 11 years [20]. Half of their patients required SP catheters with follow up imaging demonstrating complete resolution.

In partial urethral injuries, European Association of Urology (EAU) guidelines suggest either follow-up after one to two weeks of bladder drainage or a urethrogram [3]. This mirrors our findings in Table 5. Micturating cystourethrogram (MCUG) was attempted in three patients and abandoned in one of them as the catheter tip coiled upon itself in the bladder and protruded to the bulbar urethra. In the other two patients who had MCUG, the catheter was removed after imaging demonstrated no abnormalities requiring interventions.

In their retrospective study, Alcázar García et al. found a 3.3% incidence of catheter-induced urethral injury in their cohort of 181 children [21]. We believe our numbers are probably an under representation of the actual number of catheter-related injuries in our institute. The reason for that is some injuries are managed by the parent team without referring to paediatric urologists if spontaneous micturition is achieved or if they manage to catheterise uneventfully after an initial traumatic attempt. 

We had a single case of catheter-induced bulbar urethral stricture, which wouldn't permit an 8fr catheter. This was managed using cystoscopy and serial urethral dilations. More complex strictures may not be amenable to this approach and a perineal urethroplasty may be required for management [8,22-24].

False passage creation is another complication that could be acquired either with one off trial of catheterisation or with patients doing CIC for management of neuropathic bladder. Usually one to two weeks period of bladder drainage is sufficient to heal the false passage, which was our experience in two out of the three patients who developed a false passage [25-28]. However in one patient CIC couldn’t be resumed despite conservative management, and a vesicostomy was created for optimising bladder drainage. There are reports of managing those injuries with stents in adults; however, that isn’t yet popularised in paediatrics [20].

Outcomes of catheter-induced urethral injuries are generally more favourable and only a small number of patients require a more definitive approach following their initial management. Unlike the traumatic ones, which often end up with the need for internal urethrotomies or open urethroplasty.

All our patients have been males and when examining the literature, it is predominantly reported that urethral injuries are much more common in male children. This is attributed to the significantly shorter length of the urethra in girls. [13]

Most available literature discusses the acute or delayed management but doesn’t give guidance about how to follow these patients up, hence the relevance of our algorithm which doesn’t only look at acute and delayed management, but also at how to follow these patients up with a systematic approach to avoid missing post injury sequelae.

Adhering to sound urethral catheterisation technique is a key to avoiding preventable injuries [29,30].

Our cases demonstrated an inconsistent approach to investigating phases of an admission. Similarly, the follow-up was based on clinician’s preference rather than following a standard algorithm which could easily lead to missing long-term sequelae of those injuries. This inconsistency demonstrates the value of a guidance algorithm. This study has been limited by the small number of patients and its retrospective nature.

**Figure 1:**
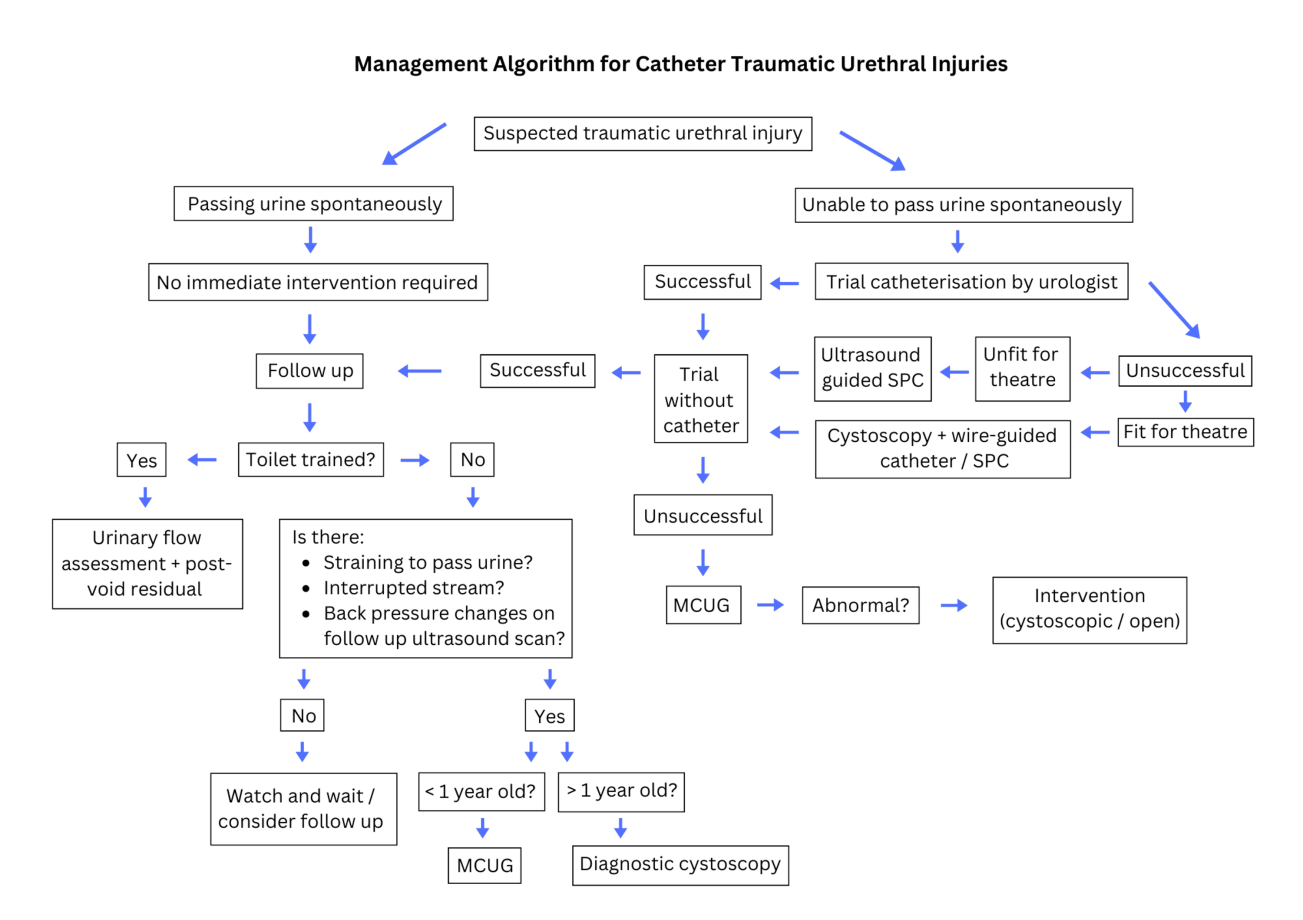
A management algorithm for catheter-induced traumatic urethral injuries This algorithm demonstrates our proposed management approach for catheter-related urethral injuries. If the child was able to urinate conservatively, our data suggested that patients can be observed and will likely have good long-term functioning. They would all require reassessment after discharge. If a child was able to participate in urinary flow assessment and post-void residual, this would be the advised investigation. If not toilet trained, we advise assessment of parental impression of whether their child’s stream is interrupted or if they strain during urination. Similarly, assessment of back-pressure changes should be done on USS. If there are no issues, then a conservative approach with further outpatient assessment would be taken. If there are issues, then we advise a MCUG assessment for children younger than one year old or cystoscopic assessment for children older than one year old. After urethral injury, if a child was unable to pass urine conservatively, then an urgent urological assessment would be appropriate. An attempt at catheterisation would be made. If unsuccessful, the patient should be assessed for theatre. If unfit, an ultrasound-guided SP catheter would be advised. If the patient was fit for theatre, then a cystoscopy + attempted wire-guided catheter or cystoscopic/USS-guided SP catheter insertion should be attempted. Once a catheter is successfully in situ and draining, the patient can be observed aiming for a TWOC in a week’s time. If the patient was then able to pass urine spontaneously, they could be managed as per the previously documented follow-up. If they were unable to pass urine, then investigating the injury with a MCUG would be appropriate. The clinician would then have suitable information to plan for catheter management and regular follow-up, or for an intervention. USS: Ultrasound scan; MCUG: Micturating cystourethrogram; TWOC: Trial without catheter; SP: Suprapubic

The references explained in our paper demonstrate a summary of available literature for paediatric catheter-induced urethral injuries. The proposed management algorithm was created using our literature review, case series and modifying BAUS algorithms for management of traumatic urethral injuries (Figure 1) [3]. Further prospective studies with larger cohort would be beneficial to statistically conclude our guidance.

## Conclusions

Catheter-related urethral injuries are common but likely to be under-reported. They are less likely to have long-term sequelae. The majority of cases do well following a period of initial bladder drainage. However, some may need further delayed interventions such as dilatation or alternate bladder drainage procedures. There is no clarity with regard to optimal management and follow-up in current literature. Our proposed algorithm for management and follow-up would help as a useful tool for management of such injuries in the paediatric population and decreases the incidence of missing long-term sequelae.
